# The Physicochemical Characteristics of Gelam Honey and Its Outcome on the Female Reproductive Tissue of Sprague–Dawley Rats: A Preliminary Study

**DOI:** 10.3390/molecules26113346

**Published:** 2021-06-02

**Authors:** Nur Hilwani Ismail, Khairul Osman, Aini Farzana Zulkefli, Mohd Helmy Mokhtar, Siti Fatimah Ibrahim

**Affiliations:** 1 Faculty of Applied Sciences, School of Biological Sciences, Universiti Teknologi MARA, Shah Alam 40450, Malaysia; hilwani@uitm.edu.my; 2 Department of Physiology, Faculty of Medicine, Universiti Kebangsaan Malaysia, Kuala Lumpur 56000, Malaysia; ainifarzana@ppukm.ukm.edu.my (A.F.Z.); helmy@ukm.edu.my (M.H.M.); 3 Centre of Diagnostic Science and Applied Health, Faculty of Health Sciences, Universiti Kebangsaan Malaysia, Bangi 43600, Malaysia; khairos@ukm.edu.my

**Keywords:** Gelam honey, phytochemical properties of honey, physicochemical profiling of honey, uterine epithelia, vagina epithelia, epithelial cell, blood glucose

## Abstract

Gelam honey (GH) is a prized natural product synthesized from the nectar of flowers from Gelam trees (*Melaleuca* sp.). Gelam is an evergreen tree species that grows in tropical regions such as Malaysia. GH is a multifloral honey with proven antioxidant and anti-inflammatory properties. However, the beneficial effect of GH on female reproductive tissue has yet to be substantiated. Herein, we investigated the effects of GH administration on the uterine and vaginal epithelial thickness of sexually mature Sprague–Dawley rats. Epithelia thickness could be an indicator of an atrophy manifesting as a symptom of a cardio syndrome. Rats were given oral doses of GH in four groups for 14 days; the lowest dose was 0.2 g GH/kg body weight (bw) rat/day and the highest dose was 8 g GH/kg bw rat/day. The physicochemical characteristics of GH were assessed through hydroxymethylfurfural and moisture content determination and sugar identification. GH attenuated the atrophy of the uterine and vaginal epithelia and increased the thickness of the endometrial stroma and endometrial surface endothelial layer. However, the dissonance observed in the effect of GH administration on the vaginal epithelium requires further investigation. Nevertheless, GH may have a strong potential in attenuating uterine and vaginal atrophies.

## 1. Introduction

**Figure A1:**
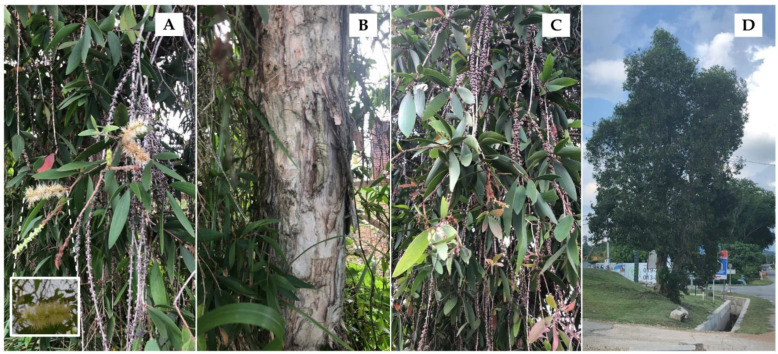
Malaysian Gelam tree (*Melaleuca* sp.) (**A**) flower stem; (**B**) bark; (**C**) seeds and (**D**) mature tree in an urban area. Images generously donated by Professor Dr. Mohd Nazip Suratman [46].

Honey is a sweet and viscid liquid produced by honey bees via a collection of floral nectar. Honey is either monofloral, i.e., originating from a single type of flora, or multifloral, i.e., originating from multiple floral sources. In Malaysia, monofloral honeys such as acacia honey and pineapple honey are produced by the *Apis mellifera* bee species [1]. Gelam honey (GH) is produced by *A. dorsata* bees and classified as a multifloral honey [2]. *A. dorsata* is a wild giant honey bee species that typically inhabits high branches in large forest trees from South to Southeast Asia [3]. GH is produced in beehives formed in Gelam trees (*Melaleuca* sp.) (Figure A1 in Appendix A), deep in Malaysia’s rainforest [4].

The increased use of honey by the local community has encouraged fraudulent activity such as adulteration with glucose on account of the lack of standard reference values developed as a standard for Malaysian honey. Only one recognized Malaysian Standard (MS 2683: 2017) for Kelulut honey is currently available [5]. This initiative was based on a national effort to provide an alternative source of income to the agriculture sector in the form of Kelulut honey. To date, however, suitable standards for GH have yet to be developed. The proposal of reference values for GH can help establish focused efforts toward developing a valid standard and safeguard the quality of Malaysia’s natural product.

Honey (and its composition) is highly variable; indeed, the components of honey are highly dependent on the geographical and botanical origin. It is considered a complex food comprising sugars, amino acids, proteins, lipids, minerals and vitamins [6,7]. The total sugar content of honey ranges from 5% to 80% and includes hexose-based sugars such as fructose, galactose, glucose, sucrose and rafinose [8]. The most prevalent monosaccharides are fructose and glucose. Honey is known as a promoter of general health with antibacterial, antioxidative, anti-inflammatory, anti-cancer, anti-adiposity and wound-healing properties [9]. The substance exerts beneficial effects on neurology with known anxiolytic, anti-nociceptive, anti-convulsant and anti-depressant effects [10]. However, the established effects of honey on the female reproductive system are limited to its use as a contraceptive measure, treatment for genital tract-related infections and pain relief during dysmenorrhea [11].

GH is a form of apitherapy and may hold potential either as a complementary therapy to hormone replacement therapy (HRT) or as an alternative therapy to HRT. Phenolic compounds in honey contribute to the latter’s antioxidative properties and have been substantiated as anti-thrombotic, anti-ischemic, antioxidant and vasorelaxant substances [12,13,14]. Thus, GH as a natural source of antioxidants with estrogenic properties is suitable for women who have been advised against using HRT because of a history of vaginal bleeding, breast cancer, gynecological cancer, myocardial infarction, stroke, heart disease, blood clots and liver disease [15,16,17].

In this study, the potential effects of GH in attenuating uterine and vaginal atrophies were investigated by analyzing the thickness of the uterine and vaginal epithelia following 14 days of GH administration in sexually mature rats.

## 2. Results

### 2.1. Physicochemical Profile of Gelam Honey for Quality Determination

**Figure 1:**
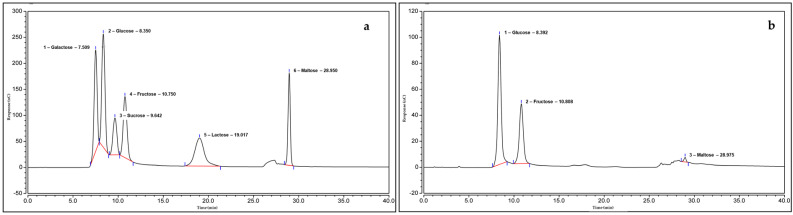
HPIC chromatograms of (**a**) the sugar standard versus (**b**) the sugar profile of Gelam honey. The retention times of the identified sugars are: glucose, 8.392 min; fructose, 10.808 min; maltose, 28.975 min.

GH is amber in color, pourable and characterized by a low viscosity; it is also sweet-smelling with a slightly acidic taste. GH had lower hydroxymethylfurfural (HMF) and moisture contents compared with Tualang honey (TH) (Table 1). The monosaccharides of the two honey types including glucose, fructose and maltose were similar (Figure 1) but the contents of each monosaccharide were generally higher in GH than in TH (Table 2). Sucrose was indetectable in GH. GH also had a higher glucose/water ratio than TH, which means the former is less able to resist crystallization during storage than the latter. All values recorded for GH complied with the suggested values in the Codex Alimentarius International Food Standards for Honey (CXS 12-1981) [18].

A mass spectral analysis of GH identified 12 semi-volatile compounds including 3-Penten-2-one,4-methyl-; 2-Pentanone,4-hydroxy-4-methyl-; 1,3-Dihydroxyacetone dimer; Benzene, (1-methylethyl)-; 1H-Pyrazole,4,5-dihydro-4,5-dimethyl-; 4H-Pyran-4-one,2,3-dihydro,3,5-dihydroxy-6-methyl-; 2-Furanmethanol; α-Methylstyrene; 5-Hydroxymethylfurfural; 2-Cyclopenten-1-one, 2-hydroxy-; Phenol and Furyl hydroxymethyl ketone (Table 3).

**Figure 2:**
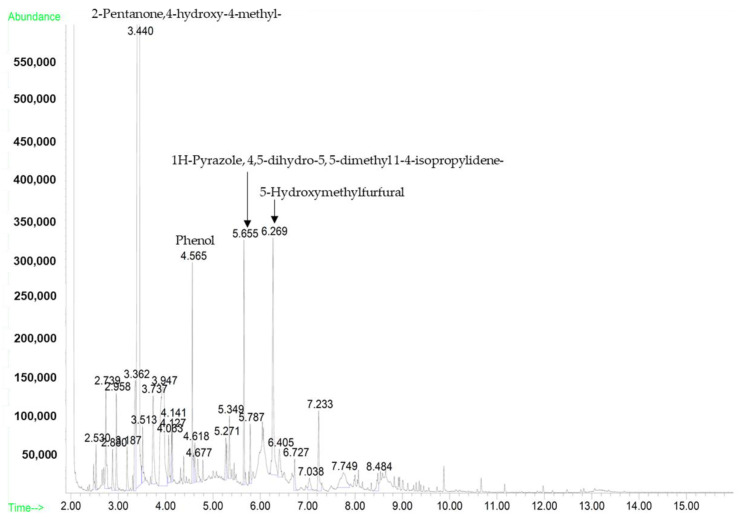
GC-MS spectrum of the four major compounds present in Gelam honey.

**Figure 3:**
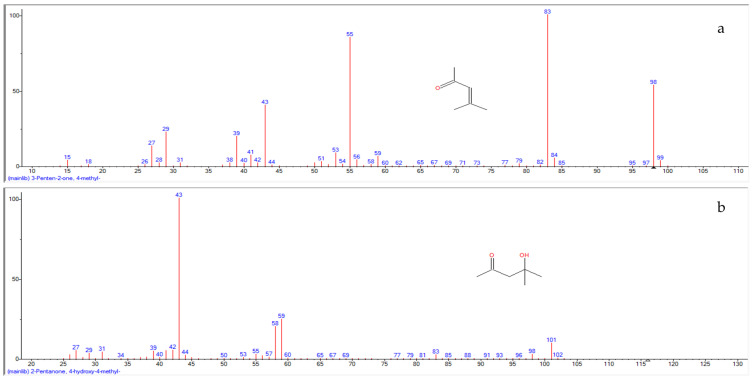

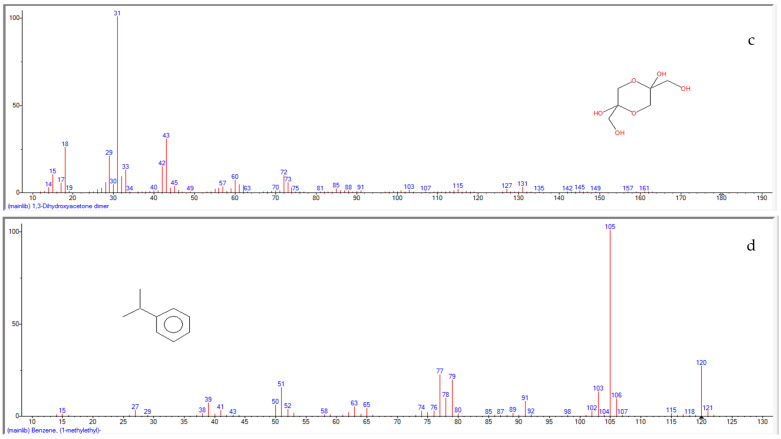

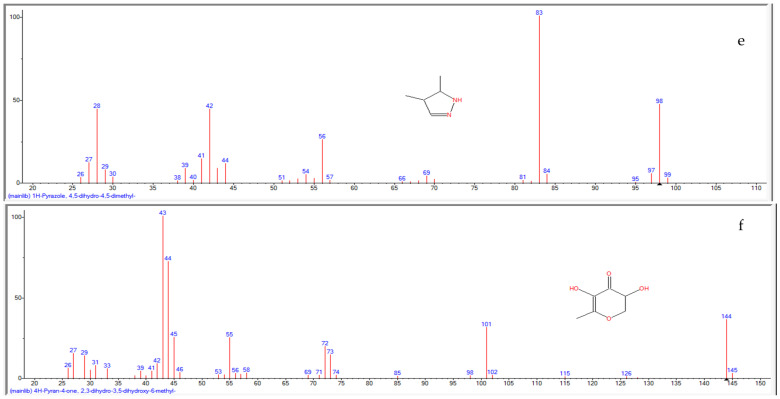

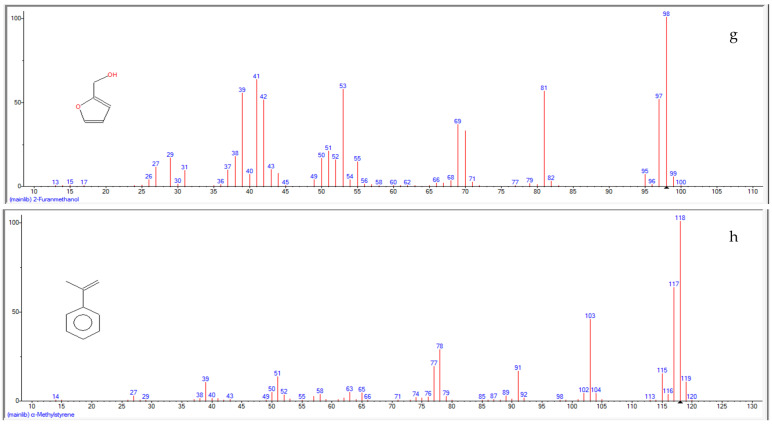

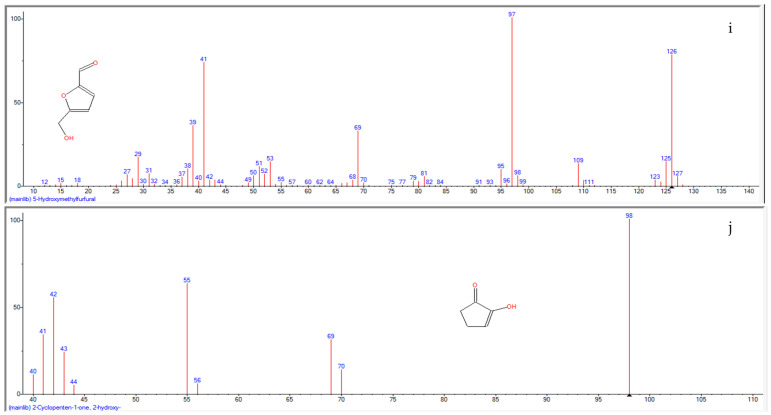

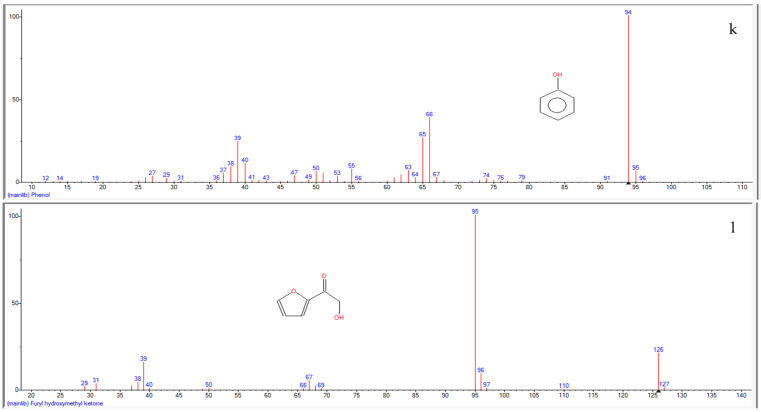
Mass spectrum of 12 semi-volatile compounds identified in Gelam honey: (**a**) 3-Penten-2-one, 4-methyl-; (**b**) 2-Pentanone, 4-hydroxy-4-methyl-; (**c**) 1,3-Dihydroxyacetone dimer; (**d**) Benzene, (1-methylethyl)-; (**e**) 1H-Pyrazole, 4,5-dihydro-4, 5-dimethyl-; (**f**) 4H-Pyran-4-one, 2,3-dihydro, 3,5-dihydroxy-6-methyl-; (**g**) 2-Furanmethanol; (**h**) α-Methylstyrene; (**i**) 5-Hydroxymethylfurfural; (**j**) 2-Cyclopenten-1-one, 2-hydroxy-; (**k**) Phenol; (**l**) Furyl hydroxymethyl ketone.

Of the 12 compounds found, four (Figure 2) were very prominently present; these compounds included 2-Pentanone,4-Hydroxy-4-methyl- (retention time (RT): 3.416 min), Phenol (RT: 4.540 min), 1H-Pyrazole,4,5-dihydro-5,5-dimethyl 1-4-isopropylidene- (RT: 5.655 min) and 5-Hydroxymethylfurfural (RT: 6.269 min). All compounds were identified by a comparison of their mass spectra with those found in the NIST mass spectral library (Figure 3a–l). A few of the compounds identified during the analysis are categorically considered phenols, furans and furanones, which are known to contribute antioxidative properties to honey.

### 2.2. Body Weight, Food Intake and FBG and Serum Hormonal Profile of Gelam Honey-Treated Rats

Throughout the experiment, the animals receiving lower doses of GH experienced a weight gain at different proportions (Table 4). GH0.2, which received 0.2 g GH/kg body weight (bw), gained the most bw with an increase of 8.28% compared with the initial weight. However, treatment with 8 g GH/kg bw/day resulted in a 6.37% bw loss during the 14-day intervention. Indeed, this treatment resulted in the greatest bw loss amongst the groups observed. The bw change between the groups was insignificant and the animals remained healthy throughout the experimental period.

**Figure 4:**
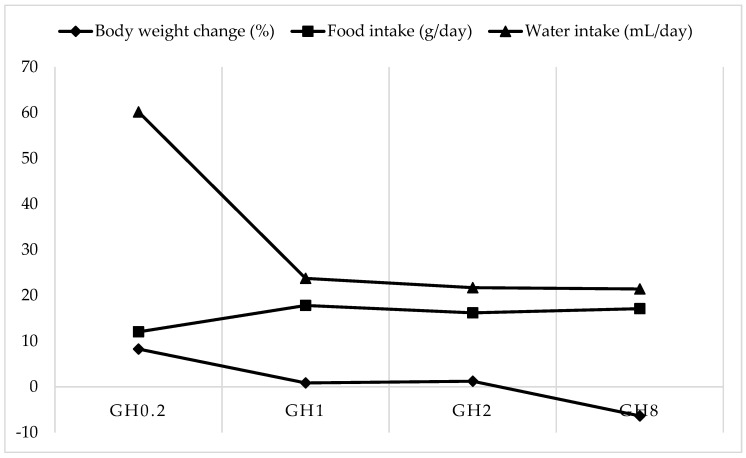
Comparison of the body weight (bw) change in relation to the food and water intake among groups. GH0.2 (0.2 g GH/kg bw/day); GH1 (1 g GH/kg bw/day); GH2 (2 g GH/kg bw/day); GH8 (8 g GH/kg bw/day).

The comparison of the body weight change in relation to the food and water intake between the groups of GH showed that a loss of body weight was not accounted for by a reduction of food intake but perhaps the reduced drinking of water (Figure 4). The food intake remained at an average of 15.79 g/day across all groups. The water intake was highest in GH0.2 at 33.39 mL/day and could have accounted for the increased percentage change in bw observed in this group.

**Figure 5:**
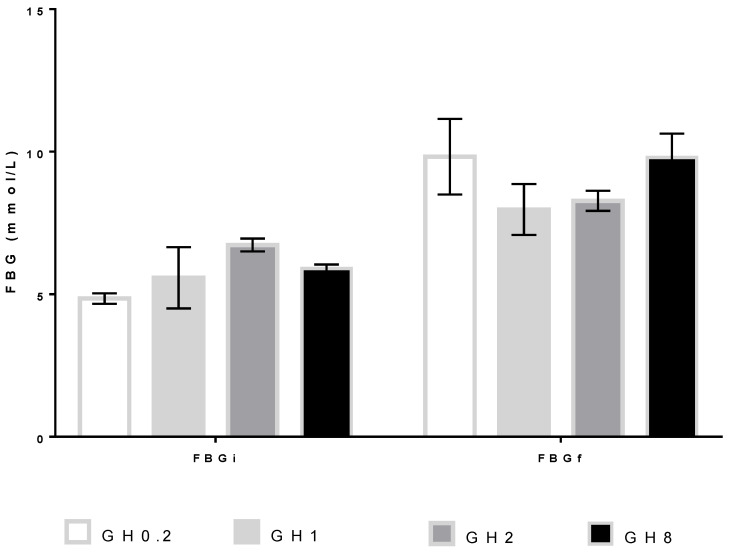
Fasting blood glucose (FBG) levels at day 0 before the administration of Gelam honey (GH; FBGi) and day 14 after the GH administration (FBGf). GH0.2 (0.2 g GH/kg bw/day); GH1 (1 g GH/kg bw/day); GH2 (2 g GH/kg bw/day); GH8 (8 g GH/kg bw/day); bw: body weight.

Whilst the bw of animals receiving GH increased, the effects of honey treatment on FBG albeit showing an incremental change were insignificant (Figure 5). The post-hoc Tukey HSD test revealed no significant difference between the different GH doses and the initial (F(3,12) = 1.896; *p* = 0.184) or final (F(3,12) = 0.893; *p* = 0.473) FBG values.

**Figure 6:**
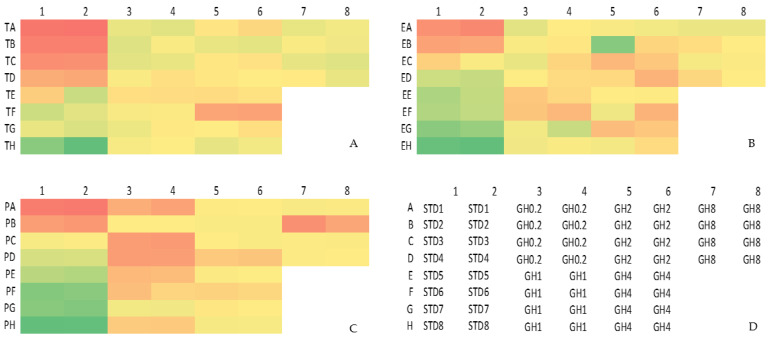
Heatmap of the measured serum hormone levels (**A**) T = testosterone, ng/mL; (**B**) E = estradiol, pg/mL; (**C**) P = progesterone, ng/mL. (**D**) Plate layout for competitive ELISA hormone assay. The red regions denote the highest concentrations whereas the green regions denote the lowest concentrations of each hormone. STD = standard. Testosterone STD1–STD8: 20, 10, 5, 2.5. 1.25, 0.625, 0.313, 0 ng/mL. Estradiol STD1–STD8: 100, 50, 25, 12.5, 6.25, 3.13, 1.56, 0 pg/mL. Progesterone STD1–STD8: 20, 10, 5, 2.5. 1.25, 0.625, 0.313, 0 ng/mL. GH0.2 (0.2 g GH/kg bw/day); GH1 (1 g GH/kg bw/day); GH2 (2 g GH/kg bw/day); GH8 (8 g GH/kg bw/day); bw: body weight.

The serum testosterone, progesterone and estradiol levels were determined to study the effects of GH administration. A post-hoc Tukey HSD test (Table 5) revealed no significant difference between the different GH doses and the hormones progesterone (F(3,12) = 1.473; *p* = 0.271), estradiol (F(3,12) = 0.294; *p* = 0.829) and testosterone (F(3,12) = 2.006; *p* = 0.167). The heatmap of the serum testosterone, progesterone and estradiol levels (Figure 6) depicts a converse relationship between the GH concentration and the measured hormone levels.

### 2.3. Uterine and Vaginal Epithelial Thickness Measurement

The uterine epithelium increased in thickness after a 14-day honey administration. The maximum thickness was obtained at a GH dose of 2.0 g/kg bw/day. The ED_50_ value was determined to be 0.14 g GH/kg bw/day. Following honey exposure, a discernible change in the thickness of the vaginal epithelium was noted. Changes in the vaginal epithelial thickness were the most prominent in GH2, which showed almost a two-fold change in thickness compared with other groups (Table 6).

**Figure 7:**
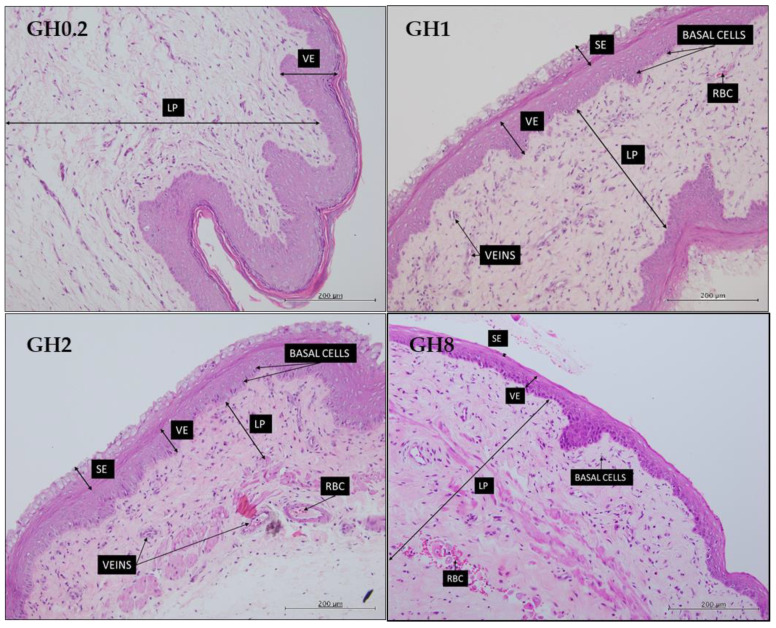
Structure of the rat vaginal epithelium following a 14-day administration of Gelam honey (GH) at different doses. The vaginal epithelium became thinner and the squamous epithelium, which makes up the mucosal layer, became less pronounced with an increasing dosage of GH. GH0.2 (0.2 g GH/kg bw/day); GH1 (1 g GH/kg bw/day); GH2 (2 g GH/kg bw/day); GH8 (8 g GH/kg bw/day). VE: vaginal epithelium; LP: lamina propia; RBC: red blood cells; SE: squamous epithelium. The tissues were stained with an H&E stain. All measurement bars are equivalent to 200 µm.

**Figure 8:**
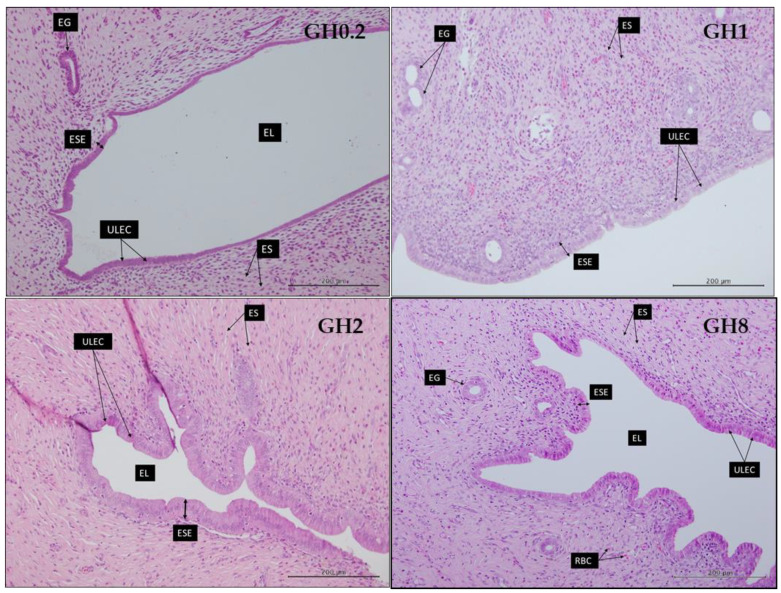
Structure of the rat uterine epithelium following a 14-day administration of Gelam honey (GH) at different dosages. The uterine epithelium initially became thicker in GH1 and GH2 but thinned with an increasing GH dosage. GH0.2 (0.2 g GH/kg bw/day); GH1 (1 g GH/kg bw/day); GH2 (2 g GH/kg bw/day); GH8 (8 g GH/kg bw/day). UE: uterine epithelium; ULEC: uterine luminal epithelial cells; ESE: endometrial surface epithelium; ES: endometrial stroma; EG: endometrial gland; EL: endometrial lumen. The issues were stained with an H&E stain. All measurement bars are equivalent to 200 µm.

The effects of GH administration on the thickness of the vaginal and uterine epithelia are shown in Figure 7 and Figure 8, respectively. Following 14 days of GH administration, the thickness of the vaginal epithelium peaked at 75.17 ± 1.91 µm in GH2. However, the thickness of the vaginal epithelium regressed to 42.43 ± 0.59 µm in GH8. Moreover, the squamous epithelium (SE), which makes up the mucosal layer of the vagina, became less pronounced with increasing the dosage of GH (Figure 7).

The uterine epithelial thickness initially became thicker in groups GH1 and GH2 but later thinned with an increasing GH dosage (Figure 8). The initial increase in the thickness of the uterine epithelium in the GH-treated groups suggested the estrogenic effect of GH on this layer.

**Figure 9:**
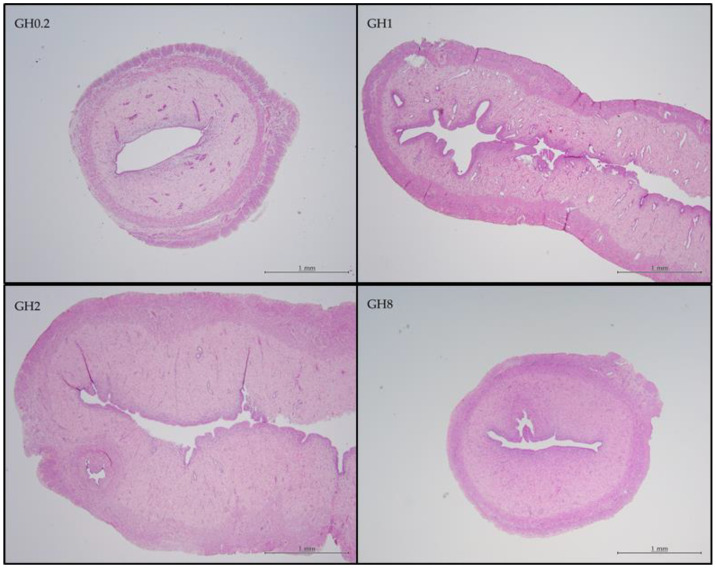
Comparison of the uterine lumen architecture following GH administration in virgin female Sprague–Dawley rats. Uterine tissues were stained with an H&E stain. All measurement bars are 1 mm. GH0.2 (0.2 g GH/kg bw/day); GH1 (1 g GH/kg bw/day); GH2 (2 g GH/kg bw/day); GH8 (8 g GH/kg bw/day); bw: body weight.

The thickness of the uterine lumen increased in the groups receiving lower GH doses on account of the thinning of the endometrial stroma (ES) layer (Figure 9). The shape of the uterine lumen in GH0.2 was more circular compared with that in other groups, thus suggesting that the ES layer was thinner in this group compared with that in the groups receiving the higher doses of GH. In addition, the endometrial surface epithelium (ESE), which makes up the uterine epithelium and is composed of cuboidal cells, appeared to become thinner with an increasing GH dosage.

The ESE in GH1 and GH2 showed columnar-shaped cells, which produced a thicker ESE layer (GH1 = 21.7381 ± 0.3142 µm; GH2 = 23.056 ± 0.379 µm). The increases in the cell density resulted in a larger surface area, which produced the undulating folds of the ESE and ultimately reduced the diameter of the endometrial lumen in the groups receiving the higher doses of GH. However, the thickness of the ESE in GH8 decreased to 22.84 ± 0.19 µm (Figure 9).

## 3. Discussion

In this study, the physicochemical properties of GH were determined. The values of the parameters were determined via the physicochemical analysis of GH (Table 7). The types of sugars (Figure 1) and major compounds (Figure 2) obtained could be regarded as reference values and a visual fingerprint for Malaysian GH. All reported values for GH conformed to the guidelines of the Codex Alimentarius International Food Standards for Honey (CXS 12-1981) [18].

The Malaysian standard MS 2683:2017 was established to support the government-led initiative of meliponiculture using stingless bees (Kelulut, *Heterotrigona itama*). Whilst *Apis* sp. produces more quantities of honey, the market demand for this type of honey is relatively lower compared with that for honey produced by stingless bees such as Kelulut honey. This demand is fueled by the belief that Kelulut honey possesses more therapeutic benefits than its counterparts including TH and GH. However, research has shown that both TH and GH possess therapeutic effects and should be recognized as a potential component of alternative medical therapy.

In this study, the physicochemical characterization of GH revealed four main chemical components as a potential fingerprint for GH. 2-Pentanone,4-hydroxy-4-methyl-, commonly known as diacetone alcohol, is a known plant metabolite that is present in cow’s milk, mung beans, soya and the human body. It belongs to the class of organic compounds known as beta-hydroxy ketones. Beta-hydroxy ketones are ketones with a beta-carbon atom containing a hydroxyl group relative to the C=O group [20]; these ketones are thus known as oxygenated hydrocarbon lipid molecules. Beta-hydroxy ketones are central to the structure of prostaglandins, an eicosanoid produced in response to mechanical, chemical or immunological stimuli [21]. Prostaglandin is known to sustain homeostatic responses such as blood flow, blood clot formation and hormone-like responses; in the uterus, it is responsible for muscle contractions for the expulsion of the uterine lining during menstruation [22,23]. Phenols are commonly known as carbolic acids and the primary source of the antioxidative properties of honey. Phenolic compounds are aromatic organic compounds associated with free radical scavenging activity, which is beneficial for the preservation of cardiovascular and neurological system health and enhancement of the immune, respiratory and gastrointestinal systems [24,25]. 1H-Pyrazole, 4,5-dihydro-5, 5-dimethyl 1-4-isopropylidene- has been identified as a chemical constituent of the essential oil from the *Tagetes minuta* flower and apricot jams [26,27]. This compound was also identified in GH and is responsible for the aroma component of the honey. The organic compound 5-HMF is a product of the Maillard reaction. HMF is the product of non-enzymatic sugar degradation. Its concentration is typically used as an indicator of honey freshness and reflects the storage time of honey or exposure to high temperatures during processing [28].

GH administration increased and prolonged the feeling of satiety and markedly reduced the thirst perception of the animals, all of which resulted in a bw loss in GH8, which received the highest dose of GH amongst the established groups. Honey is known to affect dipsogenic hormones and cause an anti-dipsogenic effect. Angiotensin II and serotonin are dipsogenic hormones that are able to increase thirst perception, resulting in an increased water intake [9,29]. However, in the present study, the rats receiving higher doses of GH experienced anti-dipsogenic effects and ultimately reduced their water intake.

Solutions with a high osmolarity can promote water movement between the intracellular and extracellular environments of the cell, which could lead to acute hypovolemia [30]. Honey, as a hyperosmotic solution, is believed to display this effect. However, the increasing doses of GH only provided an anti-dipsogenic effect and the need of the rats to consume more water decreased. GH may have increased the levels of serotonin in the rats thereby reducing the thirst perception of the animals receiving GH. The beneficial effect of GH with an increase in serotonin levels would positively impact sleeping, eating and digestion, which, in turn, would influence the general health and well-being of an individual. Honey provides neuroprotection via the antioxidant activity conferred by its components such as phenols, flavonoids, ascorbic acid, α-tocopherol, carotenoid compounds, enzymes and Maillard reaction products between the reducing sugars and the amino acids [31]. These compounds are strong radical scavengers. The ingestion of honey provides nutrition by increasing vitamin C levels, trace element absorption and the antioxidant capacity in the plasma and brain. It also increases brain-derived neurotrophins, which improve spatial memory, anxiety, behaviors and symptoms associated with depression and the stress response by lowering blood cortisol levels [32].

Fructose and glucose are the main sugar components of honey and floral honeys generally have a fructose-to-glucose ratio approximating 1 [33]. In this study, the fructose-to-glucose ratio of GH was 0.88. However, changes in the fasting blood glucose (FBG) levels of rats receiving GH were insignificant. The therapeutic effect of honey has been previously attributed to its fructose-to-glucose ratio; specifically, the hypoglycemic effect is rendered by carbohydrate metabolism, which favors fructose for first-pass removal [10]. Diabetics are widely held to benefit from honey consumption because the antioxidant compounds in honey attenuate insulin resistance [34,35].

GH0.2 recorded the highest levels of serum progesterone and testosterone but this effect was insignificant when compared with other groups (*p* > 0.05). Estradiol was highest in GH2 but the increase noted was also insignificant. However, the levels of estradiol determined across all groups were well below the reference range measured in healthy normal female rats. Honey is an estrogen modulator that can induce either an estrogenic or anti-estrogenic effect depending on the concentration used [36]. The findings from this study showed an increasing trend in the serum estradiol levels with an increasing dosage of GH. A GH dose of 0.2 g/kg bw/day may be used as a complementary HRT regimen with a known attenuating effect on estradiol and progesterone. The serum testosterone level also increased in GH0.2. Testosterone is an androgen and whilst lower levels of this hormone are expected in females, it still imparts benefits to the female reproductive function. In the female reproductive system, testosterone is converted to estradiol prior to conversion to estrogen. Testosterone is believed to alleviate the symptoms of vaginal dryness and decreased sexual contentment and libido, which are major symptoms of a genitourinary syndrome [37,38,39,40].

GH administration increased the thickness of the endometrial stroma (ES), the layer of the uterus stimulated by changes in estrogen levels. The endometrial glands present in the endometrial stroma contribute to the levels of estrogen available in the thickening tissue. GH synergistically contributes estrogenic effects on the ES; specifically, it supports the thickening of the ES layer and provides a conducive environment for the ESE to develop. A healthy ES and ESE support the production of mucous on the ESE, which is beneficial for possible implantation. However, GH administration resulted in the thinning of the VE and vaginal SE. As thinning of the SE occurs, the vagina may produce less mucous that could retain moisture and lubrication on the vaginal epithelium and provide protection against pathogenic bacteria. A major concern regarding the thinning of the vagina is dyspareunia, one of the chief complaints related to the symptoms of menopause.

The potential effects of GH in attenuating uterine and vaginal atrophies were investigated by analyzing the thickness of the uterine and vaginal epithelia following a 14-day administration of GH. The effect of GH on the VE and vaginal ESE at higher concentrations did not support its use as a potential therapy for attenuating a vaginal atrophy. However, the beneficial effect of GH administration on the uterine ES and ESE layers contributed to the thickening of the uterine layer, which meant that the substance could minimize a uterine atrophy.

The effect of GH in bilaterally ovariectomized animal models should be investigated to analyze its effect on a surgical menopause; the knowledge gained from such analyses could provide evidence of its potential in pre-menopausal and menopausal cases. GH is an apitherapy with potential applications as a complementary therapy to HRT or as an alternative therapy to HRT. However, the dissonance of the effect of GH administration on the uterine and vaginal epithelia should be considered when using GH as an apitherapy to HRT.

## 4. Materials and Methods

### 4.1. Honey Sample

The honey sample was sourced from a local experienced beekeeper in Negeri Sembilan, Malaysia (harvested from a single batch in 2019). The sample was stored in amber bottles at room temperature and protected from sunlight and heat sources until a further analysis.

### 4.2. Physicochemical Profiling of Gelam Honey for Quality Determination

#### 4.2.1. Determination of Hydroxymethylfurfural (HMF)

The HMF content was determined according to the method of [41]. Exactly 5 g of GH was dissolved in 25 mL of water and quantitatively transferred to a 50 mL volumetric flask, which was then added to 0.5 mL of Carrez solution I and 0.5 mL of Carrez solution II. The volume of the flask was increased to 50 mL with the addition of water and the resulting solution was filtered. The initial 10 mL of filtrate was discarded and two aliquots of 5 mL were placed in test tubes. The sample tube was added with 5 mL of distilled water and the reference tube was added with 5 mL of a 0.2% sodium bisulphite solution. The absorbance of the solution was determined at two wavelengths, 284 and 336 nm, by using a UV-visible spectrometer. The HMF content was calculated according to Equation (1):

HMF (mg/kg) = (A_284_) − (A_336_) × 149.7 (1)

where A_284_ was the absorbance at 284 nm, A_336_ was the absorbance at 336 nm, 149.7 was the factor and the product of the molecular weight of HMF and the mass of the sample.

#### 4.2.2. Moisture Content

The moisture content (%) and Brix (%) of honey were determined by a tri-scale automatic temperature compensation (ATC) refractometer (Tekcoplus, Hong Kong) according to the manufacturer’s instructions.

#### 4.2.3. Sugar Profiling

Sugar profiling for the GH samples was performed using the HPIC-based method as outlined in [42] and modified by [43]. In brief, 200 mg of GH was dissolved in a few milliliters of water. This solution was quantitatively transferred to a volumetric flask and filled with water to the 100 mL mark, mixed well and then filtered through a 0.02 µm nylon membrane filter (Whatman). The GH sample was injected into a Thermo Scientific™ Dionex™ ICS-5000+ system and detected using an electrochemical detector. A Dionex CarboPac PA20, Analytical (3 × 150 mm) column (Thermo Fisher Scientific, Waltham, MA, USA) was used with HPIC-grade solvents. Table 8 shows the running conditions of the HPIC system.

#### 4.2.4. Semi-Volatile Organic Compound (SVOC) Determination

GH was analyzed for SVOCs using GC/MS based on the U.S. E.P.A. 8270 method [44] with modifications [45]. The GH samples were extracted with 99.8% dichloromethane (GC grade, Merck, Darmstadt, Germany) before concentrating to the minimum injection volume. The sample was injected into the split-less inlet of an Agilent 7890B, 5977B MSD GC-MS system (Agilent Technologies, Santa Clara, CA, USA). Hydrogen served as the carrier gas and flowed at a rate of 1.0 mL/min. The SVOCs were identified by a comparison of the peaks obtained with those in the NIST library (Gaithersburg, MD, USA). The running conditions for GC and MS are indicated in Table 9.

### 4.3. Ethical Approval of Experimental Animals

The ethical review and approval of the study protocol were granted by the Universiti Kebangsaan Malaysia (UKM) Animal Ethics Committee (Ethical Approval Code FISIO/PP/2019/SITI FATIMAH/20-MAR./1000-APR.-2019-APR.-2022).

### 4.4. Animal Care and Handling

Sexually mature female Sprague–Dawley rats aged eight weeks were used in this study. The experimental animals were obtained from the institutional Laboratory Animal Research Unit (LARU), Faculty of Medicine, Universiti Kebangsaan Malaysia. All animals were individually caged, acclimatized for one week, maintained under a 12 h light/12 h dark cycle in an air-conditioned room at 25 ± 2 °C with 50–55% relative humidity and given standard food pellets and water ad libitum.

### 4.5. Experimental Design

The rats were divided into four groups (*n* = 4) corresponding with four GH concentrations: GH0.2 (0.2 g GH/kg bw/day), GH1 (1.0 g GH/kg bw/day), GH2 (2 g GH/kg bw/day) and GH8 (8 g GH/kg bw/day). Distilled water was used as a vehicle for GH and given as oral doses of 0.5 mL daily for 14 days. The measurements of bw were recorded daily. The water and food intake were recorded weekly. Throughout the treatment period, vaginal smear cytology was conducted daily to assess the reproductive cycle. The rats were sacrificed by an overdose of a ketamine-xylazine cocktail (0.1 mL/100 g rat containing 91 mg/kg ketamine and 9.1 mg/kg xylazine) administered via an intraperitonealinjection 24 h following the last administration of the treatment dose. The blood and reproductive organs (i.e., uterus and vagina) were collected for subsequent analyses.

### 4.6. Fasting Blood Glucose (FBG) Measurement

FBG was measured twice: a baseline FBGi before honey treatment and a terminal FBGf after 14 days of honey treatment. The blood for FBG determination was collected from the lateral tail vein of overnight-fasted rats and measured using a glucometer (Accu-chek Performa, Roche, Germany).

### 4.7. Serum Hormone Profile

The blood from retro-orbital bleeding was collected into serum separator tubes (BD Vacutainer SST^TM^, Becton Dickinson, Franklin Lakes, NJ, USA) and allowed to clot at room temperature prior to centrifugation at 3000× *g* for 15 min. The serum hormone levels were analyzed using a competitive enzyme-linked immunosorbent assay (ELISA) for estrogen, progesterone and testosterone in duplicate according to the manufacturer’s guidelines (Elabscience, Wuhan, China). In brief, reference wells were added with 50 µL of a biotinylated antibody solution and 50 µL of the reference standards. The test wells were added with 50 µL of a biotinylated antibody solution and 50 µL of the serum samples. This procedure was done in duplicate. The plate was then sealed and incubated for 45 min at 37 °C. The plate washing was done by aspirating the solution from each well and replacing it with 350 µL of wash buffer, which was left to soak for 1–2 min. The wash buffer was aspirated and the plate was patted dry with a paper towel. The plate washing was repeated thrice. A sum of 100 µL of horseradish peroxidase (HRP) conjugate was then added to each well and the plate was incubated for 30 min at 37 °C once more. This step was followed by a plate washing process repeated five times. The substrate-conjugating stage was a light-sensitive stage and the ELISA plate was protected from over-exposure to light. During this stage, 90 µL of the substrate reagent was added to each well and the plate was incubated for 15 min at 37 °C. Thereafter, 50 µL of a stop solution was added to each well to stop the reaction and the absorbance of the solution in the wells was immediately measured at 450 nm using a microplate reader. To ensure quality control, the stop solution was added in the same order in which the substrate solution was added. The resulting absorbance was used to calculate the exact amount of hormone present in each sample and plot a standard curve. The standard curve was generated using standard dilutions of known concentrations of the hormones. The serum hormone concentrations were determined from the generated standard curve.

### 4.8. Uterine and Vaginal Epithelial Thickness Measurement

Sections of uterine and vaginal tissue were prepared for hematoxylin and eosin (H&E) staining by processing into paraffin blocks. In brief, uterine and vaginal tissues were fixed in 10% neutral buffered formalin for 24 h and then trimmed into the appropriate sizes. The specimens were gradually dehydrated with increasing grades of ethanol (70%, 85% and 95%; Thermo Fisher Scientific, Waltham, MA, USA) for 1 h at each grade. Following dehydration, the specimens were immersed in absolute ethanol for 2 h and then cleared with xylene (Thermo Fisher Scientific, Waltham, MA, USA) twice for 1 h each time. The specimens were infiltrated with paraffin wax at 60 °C, left to equilibrate for 1 h and then solidified overnight in molds. The solidified blocks were sectioned using a microtome to produce ribbons of 5 µm. The ribbons were allowed to float in a 45 °C water bath to remove wrinkles and mounted on to glass slides by using the fishing technique. The sections were deparaffinized using xylene to allow staining with the H&E stain. The slides were stained with hematoxylin (Thermo Fisher Scientific, Waltham, MA, USA) for 5 s, rinsed with distilled water and then washed with a PBS buffer. The slides were then stained with eosin for 1 min, dehydrated twice with absolute ethanol for 3 min each time and then cleared with xylene. The slides were permanently mounted with Coverseal™-x Mounting Medium (Cancer Diagnostics Inc., Durham, NC, USA), covered with a coverslip and observed under a light microscope with a 20 × objective (Olympus BX40 Microscope with a DP27 Olympus camera and a DP-2 SAL processor). The mean uterine and vaginal epithelial thickness was determined from the measurements of six randomly chosen areas in each section using a computer-aided program (ImagePro Plus v5.0, USA).

### 4.9. Statistical Analysis

All of the data were analyzed using the IBM^®^ SPSS^®^ Statistics version 22 (IBM Corp., New York, NY, USA). Data were subjected to a one-way ANOVA with the post-hoc Tukey HSD test to evaluate the independent variables. The values were expressed as a mean ± standard error of the mean (SEM) and were considered significant at *p* < 0.05.

## Figures and Tables

**Table 1:** Hydroxymethylfurfural (HMF) and moisture contents.

	HMF (mg/100 g)	Moisture Content (%)
Gelam honey	6.9	18.75
Tualang honey	7.86 [11]	20 [19]
CODEX CXS 12-1981	<8	<21

**Table 2:** Comparison of sugar contents in Gelam honey (GH), Tualang honey (TH) and MS 2683:2017 with CODEX CXS 12-1981 standards.

	Glucose (g/100 g)	Fructose (g/100 g)	Sucrose (g/100 g)	Maltose (g/100 g)	Reducing Sugars (%)	Total Fructose and Glucose (g/100 g)	Fructose/Glucose Ratio	Glucose/Water Ratio
GH *	40.05	35.35	undetected	1.99	77.5	75.40	0.88	2.14
TH * [18]	30.0	29.6	0.6	7.9	67.5	59.6	0.99	1.5
MS 2683:2017	NS	NS	<7.5	NS	NS	<85	0.9–1.35	NS
CODEX CXS 12-1981	NS	NS	<5	NS	>65	≥60	NS	NS

NS, not specified; MS 2683:2017, quality standards for raw Kelulut honey established by the Department of Standards, Malaysia; CODEX CXS 12-1981, Codex Alimentarius International Food Standards for Honey. * GH and TH are classified as Malaysian multifloral honey.

**Table 3:** Chemical compounds identified in Gelam honey.

	RT (min)	Percentage of Total (%)	Cas. No.	MW (Da)	
**Benzene derivatives**					
Benzene, (1-methylethyl)-	4.141	2.12	98-82-8	120.1916	Aromatic, Common name: cumene
**Alcohols**					
2-Furanmethanol	3.513	18.62	98-00-0	98.0999	
**Alkenes, alkanes and alkynes**					
α-Methylstyrene	4.618	9.08	98-83-9	118.1757	Olefinic compound
**Non-aromatic ketones and aldehydes**					
2-Pentanone, 4-hydroxy-4-methyl	3.440	4.40	123-42-2	116.1583	Common name: diacetone alcohol. Beta-hydroxy ketone
1,3-Dihydroxyacetone dimer	3.947	3.85	26776-70-5	180.16	
5-Hydroxymethylfurfural	6.269	10.04	67-47-0	126.1100	
**Aromatic ketones and aldehydes**					
3-Penten-2-one, 4-methyl	2.9258	3.94	141-79-7	98.143	Olefinic compound
2-Cyclopenten-1-one, 2-hydroxy-	4.127	2.31	10493-98-8	98.0999	
**Phenols and its derivatives**					
Phenol	4.565	2.55	108-95-2	94.1112	Carbolic acid, aromatic compound
4H-Pyran-4-one, 2,3-Dihydro-3,5-dihydroxy-6-methyl-	5.787	2.67	28564-83-2	144.1253	Flavonoid
**Furans and furanone**					
Furyl hydroxymethyl ketone	5.349	3.04	17678-19-2	126.1100	
**Other hydrocarbons**					
1H-Pyrazole 4 5-dihydro-5 5-Dimethyl-4-isopropylidene-	5.655	9.09	28019-94-5	98.1463	

Da, Dalton; RT, retention time; MW, molecular weight.

**Table 4:** Summary of the final and percent change in body weight with food and water intake.

Group	Initial Body Weight (g)	Final Body Weight (g)	Body Weight Change (%)	Food Intake (g/Day)	Water Intake (mL/Day)
GH0.2	200 ± 6.976 ^a^	218.5 ± 5.752	+8.28	12.04 ^b^	33.39 ^c^
GH1	240.75 ± 12.058 ^a^	243.75 ± 14.442	+0.86	17.80 ^b^	23.75 ^c^
GH2	212.50 ± 7.399	215 ± 5.212	+1.21	16.19 ^b^	21.7 ^c^
GH8	235.25 ± 8.826	221.25 ± 5.618	‒6.37	17.11 ^b^	21.43 ^c^

Values are expressed as a mean ± SEM. Superscript letters in the same column denote significant differences within the column at *p* < 0.05. GH0.2 (0.2 g GH/kg bw/day); GH1 (1 g GH/kg bw/day); GH2 (2 g GH/kg bw/day); GH8 (8 g GH/kg bw/day); bw: body weight.

**Table 5:** Summary of the serum testosterone, progesterone and estradiol levels amongst groups receiving Gelam honey (GH).

Group	Testosterone (ng/mL)	Progesterone (ng/mL)	Estradiol (pg/mL)
GH0.2	7.05 ± 0.66	10.92 ± 0.4	16.25 ± 1.27
GH1	8.55 ± 0.62	9.63 ± 0.98	18.40 ± 4.68
GH2	8.75 ± 0.87	9.44 ± 0.5	19.95 ± 4.31
GH8	7.04 ± 0.38	9.53 ± 0.66	15.9 ± 2.26
Reference range for healthy normal female rats *	4.97–6.58	4.1–8.2	67–99

Values are presented as a mean ± SEM, *p* > 0.05 across all concentrations for all hormones. * Reference range provided in the competitive ELISA assay manual. GH0.2 (0.2 g GH/kg bw/day); GH1 (1 g GH/kg bw/day); GH2 (2 g GH/kg bw/day); GH8 (8 g GH/kg bw/day); bw: body weight.

**Table 6:** Measurements of the uterine and vaginal epithelial thickness following 14 days of Gelam honey (GH) administration to Sprague–Dawley rats (*n* = 4/group).

Groups	Uterine Epithelial Thickness (µm)	Vagina Epithelial Thickness (µm)
GH0.2	9.84 ± 0.16	47.56 ± 0.88
GH1	21.74 ± 0.31	43.09 ± 0.93
GH2	23.06 ± 0.38	75.17 ± 1.91
GH8	22.84 ± 0.19	42.43 ± 0.59

Groups reflect the dose of Gelam honey given to Sprague–Dawley rats during the 14-day treatment. GH0.2 (0.2 g/kg bw/day); GH1 (1 g/kg bw/day); GH2 (2 g/kg bw/day); GH8 (8 g/kg bw/day); bw: body weight. Values are expressed as a mean ± SEM.

**Table 7:** Physicochemical characteristics of Malaysian Gelam honey (GH) for reference values.

Physicochemical Parameters	GH
Color	amber
Hydroxymethylfurfural (mg/100 g)	6.9
Moisture content (%)	18.75
Glucose (g/100 g)	40.05
Fructose (g/100 g)	35.35
Sucrose (g/100 g)	undetected
Maltose (g/100 g)	1.99
Reducing sugars (%)	77.5
Sum of fructose and glucose (g/100 g)	75.4
Fructose/glucose ratio	0.88
Glucose/water ratio	2.14

**Table 8:** Sugar profiling conditions.

Column	Thermo Scientific Dionex CarboPac PA20 Analytical Column (3 × 150 mm)
Eluent source	Merck NaOH solution 50%
Eluent	A—200 mM NaOH, B—ultrapure water
Isocratic	5–0 min: 9 mM NaOH (equilibration) 0–24 min: 9 mM NaOH 24–24.1 min: 9–200 mM NaOH 24.1–29 min: 200 mM NaOH 29–29.1 min: 200–9 mM NaOH 29.1–40 min: 9 mM NaOH
Flow rate	0.5 mL/min
Injection volume	25 μL
Temperature	30 °C (column and detector compartments)
Backpressure	2500–3000 psi
Detection	Electrochemical detector
Background	6–10 nC
Working electrode	Gold electrode
Electrochemical cell gasket	Ultem™ 15 mil (1 mil = 0.001 inches)
Reference electrode	pH, Ag/AgCl, Ag mode
Noise	-

**Table 9:** GC/MS parameters for the determination of SVOCs in Gelam honey.

GC Parameters	
Inlet mode	Split-less
Split-less time (min)	16
Carrier gas, flow, flow rate	Helium, constant pressure 10 psi, 1.7 mL/min
Oven	50 °C, 0.1 min
Chromatographic column	30 m × 0.25 mm internal diameter × 0.5 μm film thickness DB-UI-8270D ULTRA INERT (Agilent Technologies, Santa Clara, CA, USA)
**MS Parameters**	
Transfer line temperature (°C)	300
Source temperature (°C)	230
Ionization mode	Electron ionization (EI)
Electron energy (eV)	70
Acquisition mode	Full scan 40–650 m/z
MS library	NIST MS Search 2.2 (Gaithersburg, MD, USA)

## Data Availability

The data presented in this study are available within the paper.

## Sample Availability

Samples of the compounds are available from the authors.

## References

[1] Moniruzzaman M., Khalil M.I., Sulaiman S.A., Gan S.H. (2013). Physicochemical and antioxidant properties of Malaysian honeys produced by *Apis cerana*, *Apis dorsata* and *Apis mellifera*. BMC Complementary Altern. Med..

[2] Kek S.P., Chin N.L., Yusof Y.A., Tan S.W., Chua L.S. (2017). Classification of entomological origin of honey based on its physicochemical and antioxidant properties. Int. J. Food Prop..

[3] Crane E., Resh V.H., Cardé R.T. (2009). Chapter 9-Apis Species: (Honey Bees). Encyclopedia of Insects.

[4] Azmi M.F., Abd Ghafar N., Che Hamzah J., Chua K.H., Ng S.L. (2021). The role of Gelam honey in accelerating reepithelialization of ex vivo corneal abrasion model. J. Food Biochem..

[5] Malaysian Standard (2017). MS 2683: 2017: Kelulut (Stingless Bee) Honey-Specification: Quality Requirements.

[6] Santos-Buelga C., González-Paramás A.M. (2017). Chemical Composition of Honey. Bee Products-Chemical and Biological Properties.

[7] Crane E., Resh V.H., Cardé R.T. (2009). Chapter 20-Bee products. Encyclopedia of Insects.

[8] Ball D.W. (2007). The chemical composition of honey. J. Chem. Educ..

[9] Mohd Kamal D.A., Ibrahim S.F., Kamal H., Kashim M.I.A.M., Mokhtar M.H. (2021). Physicochemical and Medicinal Properties of Tualang, Gelam and Kelulut Honeys: A Comprehensive Review. Nutrients.

[10] Mijanur Rahman M., Gan S.H., Khalil M. (2014). Neurological effects of honey: Current and future prospects. Evid. Based Complement. Altern. Med..

[11] Ismail N.H., Ibrahim S.F., Jaffar F.H.F., Mokhtar M.H., Chin K.Y., Osman K. (2021). Augmentation of the Female Reproductive System Using Honey: A Mini Systematic Review. Molecules.

[12] Khalil M.I., Sulaiman S.A., Gan S.H. (2010). High 5-hydroxymethylfurfural concentrations are found in Malaysian honey samples stored for more than one year. Food Chem. Toxicol..

[13] Khalil M.L., Sulaiman S.A. (2010). The potential role of honey and its polyphenols in preventing heart disease: A review. Afr. J. Tradit. Complement. Altern. Med..

[14] Yao L.K., Razak S.L., Ismail N., Fai N.C., Asgar M.H., Sharif N.M., Aan G.J., Jubri Z. (2011). Malaysian gelam honey reduces oxidative damage and modulates antioxidant enzyme activities in young and middle aged rats. J. Med. Plant Res..

[15] Ibeanu O., Modesitt S.C., Ducie J., Von Gruenigen V., Agueh M., Fader A.N. (2011). Hormone replacement therapy in gynecologic cancer survivors: Why not?. Gynecol. Oncol..

[16] Cardoso C.G., Rosas F.C., Oneda B., Labes E., Tinucci T., Abrahão S.B., da Fonseca A.M., Mion D., de Moraes Forjaz C.L. (2011). Aerobic training abolishes ambulatory blood pressure increase induced by estrogen therapy: A double blind randomized clinical trial. Maturitas.

[17] Cagnacci A., Venier M. (2019). The controversial history of hormone replacement therapy. Medicina.

[18] Codex Alimentarius (1981). International Standard for Honey CXS 12-19811 Adopted in 1981.

[19] Erejuwa O.O., Sulaiman S.A., Wahab M.S., Sirajudeen K.N.S., Salleh M.M., Gurtu S. (2010). Antioxidant protection of Malaysian tualang honey in pancreas of normal and streptozotocin-induced diabetic rats. Annales D’endocrinologie.

[20] NCBI (National Center for Biotechnology Information) (2021). PubChem Compound Summary for CID 31256, Diacetone Alcohol. https://pubchem.ncbi.nlm.nih.gov/compound/Diacetone-alcohol.

[21] Wishart D.S., Feunang Y.D., Marcu A., Guo A.C., Liang K., Vázquez-Fresno R., Sajed T., Johnson D., Li C., Karu N. (2018). Metabocard for Diacetone alcohol (HMDB0031511). HMDB 4.0—The Human Metabolome Database for 2018. Nucleic Acids Res..

[22] Wånggren K., Stavreus-Evers A., Olsson C., Andersson E., Gemzell-Danielsson K. (2008). Regulation of muscular contractions in the human Fallopian tube through prostaglandins and progestagens. Hum. Reprod..

[23] Ricciotti E., FitzGerald G.A. (2011). Prostaglandins and inflammation. Arterioscler. Thromb. Vasc. Biol..

[24] Cianciosi D., Forbes-Hernández T.Y., Afrin S., Gasparrini M., Reboredo-Rodriguez P., Manna P.P., Zhang J., Bravo Lamas L., Martínez Flórez S., Agudo Toyos P. (2018). Phenolic compounds in honey and their associated health benefits: A review. Molecules.

[25] Olas B. (2020). Honey and its phenolic compounds as an effective natural medicine for cardiovascular diseases in humans?. Nutrients.

[26] Igwaran A., Iweriebor B.C., Okoh S.O., Nwodo U.U., Obi L.C., Okoh A.I. (2017). Chemical constituents, antibacterial and antioxidant properties of the essential oil flower of *Tagetes minuta* grown in Cala community Eastern Cape, South Africa. BMC Complement. Altern. Med..

[27] Núñez-Carmona E., Abbatangelo M., Zottele I., Piccoli P., Tamanini A., Comini E., Sberveglieri G., Sberveglieri V. (2019). Nanomaterial gas sensors for online monitoring system of fruit jams. Foods.

[28] Shapla U.M., Solayman M., Alam N., Khalil M.I., Gan S.H. (2018). 5-Hydroxymethylfurfural (HMF) levels in honey and other food products: Effects on bees and human health. Chem. Cent. J..

[29] Katayama Y., Takei Y., Kusakabe M., Sakamoto T. (2019). Hormonal regulation of thirst in the amphibious ray-finned fish suggests the requirement for terrestrialization during evolution. Sci. Rep..

[30] Epstein A.N., Hsiao S., Peters G., Fitzsimons J.T., Peters-Haefeli L. (1975). Angiotensin as Dipsogen. Control Mechanisms of Drinking.

[31] Ali A.M., Hendawy A.O. (2018). Bee honey as a potentially effective treatment for depression: A review of clinical and preclinical findings. JOJ Nurs. Health Care.

[32] Ali A.M., Kunugi H. (2019). Bee honey protects astrocytes against oxidative stress: A preliminary in vitro investigation. Neuropsychopharmacol. Rep..

[33] Kirs E., Pall R., Martverk K., Laos K. (2011). Physicochemical and melissopalynological characterization of Estonian summer honeys. Procedia Food Sci..

[34] Erejuwa O.O., Sulaiman S.A., Ab Wahab M.S. (2012). Honey—A Novel Antidiabetic Agent. Int. J. Biol. Sci..

[35] Ramli E.S.M., Sukalingam K., Kamaruzzaman M.A., Soelaiman I.N., Pang K.L., Chin K.Y. (2021). Direct and Indirect Effect of Honey as a Functional Food Against Metabolic Syndrome and Its Skeletal Complications. Diabetes Metab. Syndr. Obes. Targets Ther..

[36] Erejuwa O.O., Sulaiman S.A., Wahab M.S.A. (2014). Effects of honey and its mechanisms of action on the development and progression of cancer. Molecules.

[37] Abraham G.E. (1974). Ovarian and adrenal contribution to peripheral androgens during the menstrual cycle. J. Clin. Endocrinol. Metab..

[38] Burger H.G. (2002). Androgen production in women. Fertil. Steril..

[39] Davison S.L., Bell R., Donath S., Montalto J.G., Davis S.R. (2005). Androgen levels in adult females: Changes with age, menopause, and oophorectomy. J. Clin. Endocrinol. Metab..

[40] Angelou K., Grigoriadis T., Diakosavvas M., Zacharakis D., Athanasiou S. (2020). The genitourinary syndrome of menopause: An overview of the recent data. Cureus.

[41] White J.W. (1979). Spectrophotometric method for hydroxymethylfurfural in honey. J. AOAC Int..

[42] Bogdanov S., Lüllmann C., Martin P., von der Ohe W., Russmann H., Vorwohl G., Oddo L.P., Sabatini A.G., Marcazzan G.L., Piro R. (1999). Honey quality and international regulatory standards: Review by the International Honey Commission. Bee World.

[43] Huang B., Hu J., Rohrer J. (2016). Determination of carbohydrates in kombucha using HPAE-PAD. Abstracts of Papers of the American Chemical Society.

[44] U.S. EPA (2014). Method 8270E (SW-846): Semivolatile Organic Compounds by Gas Chromatography/Mass Spectrometry (GC/MS).

[45] Moniruzzaman M., Rodríguez I., Ramil M., Cela R., Sulaiman S.A., Gan S.H. (2014). Assessment of gas chromatography time-of-flight accurate mass spectrometry for identification of volatile and semi-volatile compounds in honey. Talanta.

[46] Suratman M.N. (2021). Personal communication.

